# Alternate SlyA and H-NS nucleoprotein complexes control *hlyE* expression in *Escherichia coli* K-12

**DOI:** 10.1111/j.1365-2958.2007.05950.x

**Published:** 2007-09-24

**Authors:** James K Lithgow, Fouzia Haider, Ian S Roberts, Jeffrey Green

**Affiliations:** 1 Department of Molecular Biology and Biotechnology, The University of Sheffield Western Bank, Sheffield S10 2TN, UK.; 2 1.800 Stopford Building, Faculty of Life Sciences, University of Manchester Oxford Road, Manchester M13 9PT, UK.

## Abstract

Haemolysin E is a cytolytic pore-forming toxin found in several *Escherichia coli* and *Salmonella enterica* strains. Expression of *hlyE* is repressed by the global regulator H-NS (histone-like nucleoid structuring protein), but can be activated by the regulator SlyA. Expression of a chromosomal *hlyE–lacZ* fusion in an *E. coli slyA* mutant was reduced to 60% of the wild-type level confirming a positive role for SlyA. DNase I footprint analysis revealed the presence of two separate SlyA binding sites, one located upstream, the other downstream of the *hlyE* transcriptional start site. These sites overlap AT-rich H-NS binding sites. Footprint and gel shift data showed that whereas H-NS prevented binding of RNA polymerase (RNAP) at the *hlyE* promoter (P*hlyE*), SlyA allowed binding of RNAP, but inhibited binding of H-NS. Accordingly, *in vitro* transcription analyses showed that addition of SlyA protein relieved H-NS-mediated repression of *hlyE*. Based on these observations a model for SlyA/H-NS regulation of *hlyE* expression is proposed in which the relative concentrations of SlyA and H-NS govern the nature of the nucleoprotein complexes formed at P*hlyE*. When H-NS is dominant RNAP binding is inhibited and *hlyE* expression is silenced; when SlyA is dominant H-NS binding is inhibited allowing RNAP access to the promoter facilitating *hlyE* transcription.

## Introduction

Cytolytic toxins are major virulence factors secreted by bacterial pathogens. The most extensively studied of these is haemolysin A, a 110 kDa protein belonging to the family of RTX ‘repeat toxins’, which is secreted by a specific type I secretion system and associates with target cell membranes where it is thought to form pores (Felmlee *et al*., 1985; Bhakdi *et al*., 1989; Welch *et al*., 1992). Another unrelated pore-forming toxin, haemolysin E, designated HlyE, ClyA or SheA, is a 34 kDa protein identified in *Escherichia coli* and *Salmonella enterica* serovars Typhi and Paratyphi A (Ludwig *et al*., 1995; 1999; Oscarsson *et al*., 1996; 2002; del Castillo *et al*., 1997; Fernandez *et al*., 1998; Atkins *et al*., 2000; Wallace *et al*., 2000; von Rhein *et al*., 2006). The pore structure of HlyE remains controversial in that separate studies have suggested that it can form either predominantly octomeric or predominantly 13-meric rings that can insert into cell membranes (Eifler *et al*., 2006; Tzokov *et al*., 2006). HlyE export is not yet fully understood, although it is known that HlyE accumulates in the periplasm of *E. coli* independently of type I, II, III, IV and V secretion systems, and its subsequent release is mediated, at least in part, by membrane blebbing (Wai *et al*., 2003; Wyborn *et al*., 2004a). It has been shown that HlyE affects Ca^2+^ signalling in epithelial cells, induces apoptosis in human and mouse macrophages, and causes haemolysis of human, rabbit, sheep and horse erythrocytes (Oscarsson *et al*., 1999; Lai *et al*., 2000; Soderblom *et al*., 2002; 2005).

Several different types of *E. coli*, including Shiga toxin-producing (O157:H7), enteroinvasive, enteroaggregative, enterotoxigenic and avian strains, have been shown to carry functional copies of the *hlyE* gene (del Castillo *et al*., 2000; Ludwig *et al*., 2004; Wyborn *et al*., 2004a; Kerenyi *et al*., 2005; McPeake *et al*., 2005). In contrast, some other enteropathogenic strains have non-functional *hlyE* genes containing frameshift mutations, and strains isolated from extraintestinal (e.g. uropathogenic or newborn meningitis-associated) infections harbour non-functional copies of *hlyE* with chromosomal deletions (Ludwig *et al*., 2004). It has been suggested that due to interference between the mechanisms regulating production of HlyA and HlyE, there is possible incompatibility between *hlyA* and *hlyE* in the *E. coli* chromosome (Kerenyi *et al*., 2005). A functional *hlyE* gene is also present in non-pathogenic *E. coli* K-12, but under normal laboratory conditions HlyE is not produced at phenotypically detectable levels due to repression by the nucleoid-associated regulatory protein H-NS (histone-like nucleoid structuring protein) (Ludwig *et al*., 1999; Westermark *et al*., 2000). HlyE only usually causes haemolysis when its gene is carried on high-copy-number plasmids (del Castillo *et al*., 1997), in derepressed *hns* mutants (Westermark *et al*., 2000) or when certain transcriptional regulators, such as SlyA from *E. coli* or *S. enterica* serovar Typhimurium (*S*. Typhimurium), are overexpressed (Ludwig *et al*., 1995; del Castillo *et al*., 1997).

SlyA is a member of the family of ‘winged-helix’ transcription factors that includes MarR from *E. coli*, RovA from *Yersinia* spp. and PecS from *Erwinia chrysanthemi* (Reverchon *et al*., 1994; Sulavik *et al*., 1995; Revell and Miller, 2000; Heroven *et al*., 2004; Cathelyn *et al*., 2006; Ellison and Miller, 2006; Wilkinson and Grove, 2006). SlyA regulates the expression of a large number of *S.* Typhimurium genes, the majority of which are predicted to encode membrane, periplasmic or secreted proteins, suggesting that a major role of SlyA is to alter the cell envelope during stationary phase and in the intracellular environment of host cells (Spory *et al*., 2002; Stapleton *et al*., 2002; Navarre *et al*., 2005). SlyA is implicated in virulence, survival in mouse macrophages, resistance to oxidative stress and resistance to antimicrobial peptides (Libby *et al*., 1994; Daniels *et al*., 1996; Buchmeier *et al*., 1997; Spory *et al*., 2002; Shi *et al*., 2004; Linehan *et al.*, 2005). Analysis of the transcriptome of a *S.* Typhimurium *slyA* mutant revealed that many *slyA*-dependent genes are also controlled by the magnesium-sensitive PhoP/PhoQ regulatory system (Navarre *et al*., 2005).

SlyA forms a dimer and has a DNA-binding domain containing a DNA-recognition helix flanked by a β-strand wing (Alekshun *et al*., 2001; Wu *et al*., 2003; Okada *et al*., 2007). The DNA-binding motifs are separated by a channel that allows contact with both the minor and major grooves of the DNA helix (Okada *et al*., 2007). The *S.* Typhimurium SlyA dimer (32 kDa) recognizes the palindromic DNA consensus sequence TTAGCAAGCTAA (Stapleton *et al*., 2002). Although the RovA protein in *Yersinia pseudotuberculosis* has been shown to directly activate transcription by stimulating RNA polymerase (RNAP) in the absence of other accessory factors, it is thought that SlyA-type proteins generally activate transcription *indirectly*, by binding to DNA and modulating the activity of other factors such as PhoP or the nucleoid-associated protein H-NS (Heroven *et al*., 2004; Navarre *et al*., 2005; Tran *et al*., 2005). Some members of the MarR subfamily interact with small ligands to alter the function of the protein, but as yet no such molecular signal has been identified for SlyA (Kenney, 2002; Wilkinson and Grove, 2006).

H-NS is a 15 kDa protein that acts as a regulator of more than 200 *E. coli* genes mostly related to adaptation to environmental stress (Drlica and Rouviere-Yaniv, 1987; Hulton *et al*., 1990; Atlung and Ingmer, 1997). H-NS can bind to DNA and form higher-order oligomeric H-NS complexes in a concentration-dependent manner, changing DNA topology and interfering with the action of other transcription factors (Tupper *et al*., 1994; Esposito *et al*., 2002; Rimsky, 2004). It has been shown that H-NS binds to the upstream and downstream regions of the *hlyE* promoter (P*hlyE*) in *E. coli* and is involved in silencing *hlyE* expression (Westermark *et al*., 2000). Further investigation showed that the P*hlyE* contained overlapping high-affinity binding sites for both H-NS and SlyA (Wyborn *et al*., 2004b). In addition, P*hlyE* was characterized as a class I promoter that could be activated by the oxygen-responsive regulator FNR and/or the glucose-responsive regulator CRP (Green and Baldwin, 1997; Ralph *et al*., 1998; Westermark *et al*., 2000). In this study, using *in vivo* and *in vitro* analyses the interactions between H-NS, SlyA and RNAP at the P*hlyE* were examined to elucidate the mechanism whereby SlyA can antagonize H-NS-mediated repression of *hlyE* transcription.

## Results

### Effect of *hns* and *slyA* mutations on chromosomal *hlyE–lacZ* expression *in vivo*

The level of *hlyE* expression is increased in an *E. coli hns* mutant (Westermark *et al*., 2000; Wyborn *et al*., 2004b). Expression of *hlyE–lacZ* carried on a multicopy plasmid is enhanced by introduction of multicopy *slyA* in a parent strain but not in an *hns* mutant, suggesting that SlyA has no intrinsic activator role, but that it probably relieves H-NS-mediated repression of *hlyE* transcription (Wyborn *et al*., 2004b). To discount the possibility that previous results were affected by plasmid copy number and regulator titration the effects of *hns* and *slyA* mutations on expression of a chromosomal *hlyE–lacZ* fusion were determined (Table 1). Aerobic cultures supplemented with 0.2% glucose were used to minimize the activities of FNR and CRP proteins. At 20°C, β-galactosidase activity of the *hns* mutant cultures was 1.8-fold higher than of the cultures of the parent strain, confirming the repressive effect of H-NS on *hlyE–lacZ* expression. Expression of *hlyE* in a *slyA* mutant was reduced to 60% of that of the parent, demonstrating a positive effect of *slyA* in the presence of functional *hns* gene. In the *hns slyA* double mutant, *hlyE* expression was twofold greater than in the parent, and slightly higher than the *hns* single mutant, suggesting that *slyA* has a small negative effect on *hlyE* expressionin the absence of H-NS. At 37°C, *hlyE–lacZ* expression was ∼25% lower than that observed at 20°C for theparental, *hns*, and *hns slyA* strains (Table 1). However, expression of *hlyE* in the *slyA* mutant was similar at both temperatures. These data support the hypothesis that SlyA relieves H-NS-mediated repression of *hlyE* expression.

**Table 1:** Effect of *hns* and *slyA* mutations on expression of a chromosomal *hlyE–lacZ* fusion.

	Strain^a^	M182	hns	slyA	hns slyA
20°C	β-Galactosidase activity (Miller units)^b^	1274 ± 48	2353 ± 164	709 ± 36	2577 ± 83
	Ratio^c^		1.8	0.6	2.0
37°C	β-Galactosidase activity (Miller units)^b^	940 ± 104	1729 ± 165	721 ± 38	1824 ± 47
	Ratio^c^		1.8	0.8	1.9

a Strains with the single-copy chromosomal *hlyE–lacZ* reporter fusion were grown in aerobic conditions with 0.2% glucose for 16 h at 20°C or 37°C: M182, parent JRG5580; *hns*, JRG5582; *slyA*, JRG5581; *hns slyA*, JRG5583.

b Means (*n* = 3) and standard deviations are shown.

c Ratio of mutant strain β-galactosidase activity/M182 parent strain activity.

### SlyA binds at two separate sites at the *hlyE* promoter and inhibits H-NS binding

**Fig. 1:**
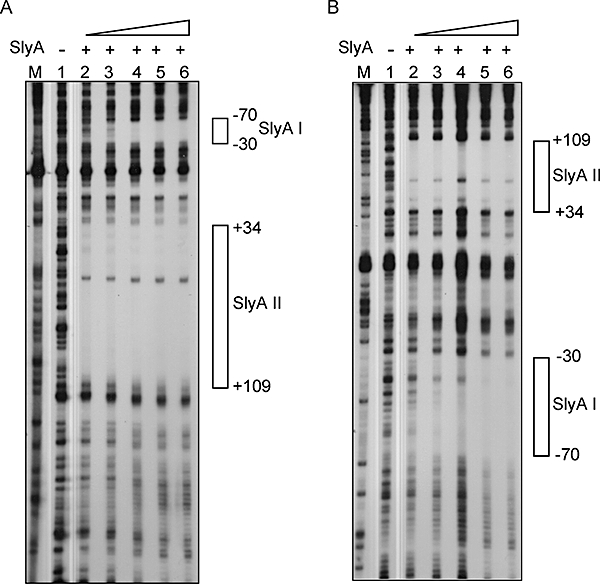
Identification of two SlyA protected regions of the *hlyE* promoter. In (A) a radiolabelled 393 bp DNA fragment (−171 to +222 relative to *hlyE* transcript start) was used in DNase I protection assays with SlyA protein. Lane M, Maxam–Gilbert G-track; lane 1, no protein; lanes 2–6, SlyA (0.3, 0.6, 1.2, 2.4 and 3.6 μM respectively). In (B) the DNA fragment was labelled on the opposite strand. Regions of SlyA protection (open boxes) and numbering relative to the *hlyE* transcript start site are shown.

Previous *in vitro* DNase I protection analysis with P*hlyE* identified a SlyA footprint from −70 to −30 relative to the *hlyE* transcription start site, reflecting the presence of two separate palindromic SlyA-recognition sequences in this upstream region, these being SlyA Ia (^−61^**TTA**T**CA**TAT**TAA**^−50^) and SlyA Ib (^−50^A**TAG**A**AA**TAA**A**G^−39^) (consensus-matching bases in bold) (Wyborn *et al*., 2004b). In this work, using a P*hlyE* DNA fragment extending from −171 to +222, we have observed another SlyA binding site (named SlyA II) in the region downstream of the transcript start site. In the presence of SlyA, protection of P*hlyE* was observed from −70 to −30 (corresponding to the previously described SlyA I site) and also in the region downstream of the transcript start site from +34 to +109 (Fig. 1). The crystal structures of *E. coli* MarR and *Enterococcus faecalis* SlyA-like proteins show their cross-sections are ∼70 Å, suggesting they would protect ∼20 bp of DNA (Alekshun *et al*., 2001; Wu *et al*., 2003). This suggests the presence of two SlyA dimers at the SlyA I site and at least three dimers at the SlyA II site. The downstream site, SlyA II, was protected in the presence of SlyA at concentrations of 0.3 μM and above, whereas the SlyA I site was only partially protected in the presence of 0.6 μM and fully protected with 1.2 μM SlyA (Fig. 1), suggesting that the SlyA protein has a higher affinity for the SlyA II site.

Wyborn *et al*. (2004b) described two regions of H-NS binding at the P*hlyE* using 1 μM H-NS which extended from −75 to −37 (H-NS I) and from −22 to +11 (H-NS II). At higher concentrations up to 9.6 μM, H-NS was shown to interact with a larger region of P*hlyE* extending from −137 to +182 (Westermark *et al*., 2000). Taken together these data suggested that at lower concentrations H-NS may bind at AT-rich specific nucleation sites, and at higher concentrations, multiple H-NS proteins oligomerize to occupy both the upstream and downstream regions of P*hlyE*. To test the hypothesis that SlyA can modulate the interaction of H-NS with P*hlyE*, footprinting analysis was performed using combinations of H-NS and SlyA protein.

**Fig. 2:**
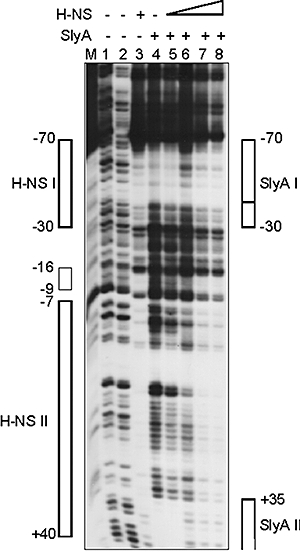
H-NS competes with SlyA for binding at the *hlyE* promoter. DNase I protection assays at the *hlyE* promoter in the presence of SlyA and H-NS. Lane M, Maxam–Gilbert G-track; lanes 1 and 2, no protein; lane 3, H-NS (2.4 μM); lane 4, SlyA (2 μM); lanes 5–8, H-NS (0.1, 1.2, 2.4 and 3.6 μM respectively) with fixed amount of SlyA (2 μM). Regions of protection (open boxes) and numbering relative to the *hlyE* transcript start site are shown.

Using 2.4 μM H-NS, regions of P*hlyE* protection were observed from −70 to −30 [corresponding to the H-NS I site found by Wyborn *et al*. (2004b)], −16 to −9, and −7 to +40 (corresponding approximately to the H-NS II site) (Fig. 2, lane 3). The H-NS footprint was also characterized by hypersensitive bases at −30, −17 and −8. With 2 μM SlyA, protection was observed at −70 to −38 (SlyA I) and from +35 to beyond the end of the autoradiographs (SlyA II), with a hypersensitive region from −30 to −9 (Fig. 2, lane 4). In reactions where 0.1 μM H-NS was added to the SlyA:P*hlyE* complex, both SlyA I and II sites remained protected (Fig. 2, lane 5). On addition of 1.2 μM H-NS, protection of the SlyA I and II sites was abolished, and replaced by weak protection of the H-NS II site, but no apparent protection of H-NS I (Fig. 2, lane 6). Furthermore, the pattern of hypersensitive bases from −30 to −9 remained, suggesting that the addition of 1.2 μM H-NS allowed some SlyA to associate with the complex, and that H-NS was unable to fully interact with P*hlyE*. In reactions where 2.4 or 3.6 μM H-NS were added, the pattern of protection observed resembled the footprint made by the H-NS protein alone, with protection at both H-NS I and II sites (Fig. 2, lanes 7 and 8).

**Fig. 3:**
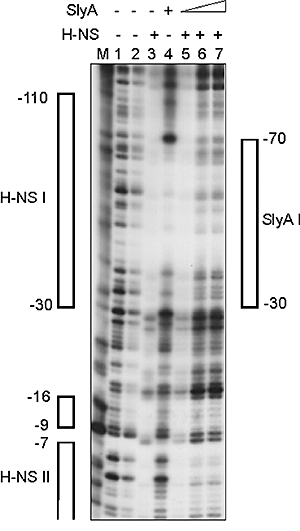
SlyA remodels a P*hlyE*:H-NS nucleoprotein complex. DNase I protection assays of the P*hlyE*:H-NS complex in the presence of increasing concentrations of SlyA. Lane M, Maxam–Gilbert G-track; lanes 1 and 2, no protein; lane 3, H-NS (2.4 μM); lane 4, H-NS (2.4 μM); lanes 5–7, SlyA (0.1, 0.5 and 1 μM respectively) with fixed amount of H-NS (2.4 μM). Regions of protection (open boxes) and numbering relative to the *hlyE* transcript start site are shown.

In reactions where 0.1 μM SlyA was added to P*hlyE* before addition of 2.5 μM H-NS (Fig. 3, lane 5), the resulting footprint resembled that produced by H-NS alone (Fig. 3, lane 3). When 0.5 μM or 1 μM SlyA was added before H-NS, the pronounced H-NS footprint was absent, and hypersensitive sites were present at −91, −30, −16 (Fig. 3, lanes 6 and 7). Therefore, in the presence of a high level of H-NS, addition of a low level of SlyA did not affect the binding of H-NS at P*hlyE*, but when the level of SlyA was increased, a unique pattern of protection was obtained which is distinct from, yet shares some features of, the footprints made by H-NS or SlyA alone.

Overall, the footprinting data suggested that a P*hlyE*:SlyA complex is formed when SlyA binds at specific sites in the upstream and downstream regions of the promoter, and when present at a high enough concentrations, SlyA prevents the binding of low levels of H-NS at the H-NS I and H-NS II sites. At intermediate concentrations of H-NS, the pattern of protection and hypersensitive bases suggests that the structure of the P*hlyE*:SlyA complex is altered, but H-NS is unable to fully interact with P*hlyE*. When H-NS is present at a higher concentration it can displace SlyA from SlyA sites I and II and thus occupy the H-NS I and II sites enabling the formation of higher-order H-NS complexes.

### Interaction of SlyA, H-NS and RNAP at the *hlyE* promoter

It has been suggested that H-NS binding renders P*hlyE* inaccessible to RNAP (Westermark *et al*., 2000). Whereas previous work analysed the interaction of either H-NS or SlyA with P*hlyE*, in this study electrophoretic mobility shift assays (EMSA) were used to examine the interaction of mixtures of H-NS, SlyA and RNAP with P*hlyE*.

**Fig. 4:**
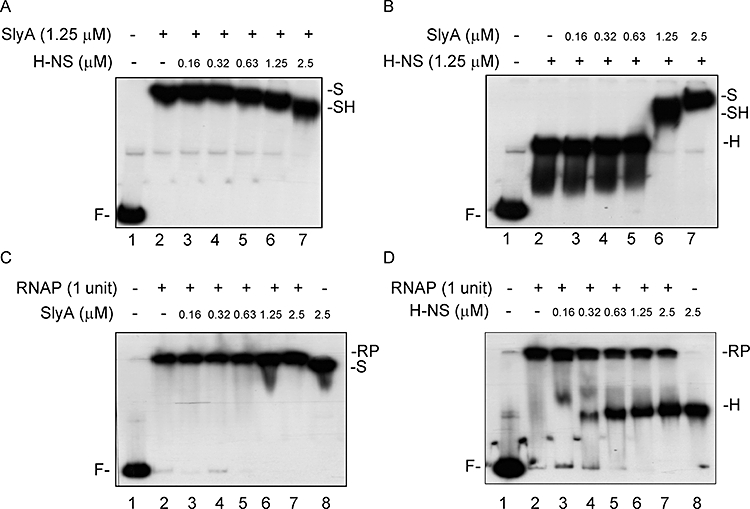
Interaction of SlyA, H-NS and RNAP with *hlyE* promoter DNA. Electrophoretic mobility shift assays were carried out in 4% acrylamide/1 × TBE gels using 20 ng of a radiolabelled 244 bp DNA fragment (−171 to +73 relative to the *hlyE* transcript start) combined with varying amounts of protein: (A) fixed amount (1.25 μM) of SlyA with increasing level of H-NS; (B) fixed amount of H-NS (1.25 μM) with increasing level of SlyA; (C) 1 unit of RNAP with increasing level of SlyA; (D) 1 unit of RNAP with increasing level of H-NS. Protein concentrations are shown in μM. F, free DNA; S, SlyA:DNA complex; H, H-NS:DNA complex; SH, SlyA:H-NS:DNA complex; RP, RNAP:DNA complex.

A SlyA:P*hlyE* complex with significantly retarded mobility was formed on addition of 1.25 μM SlyA to P*hlyE* DNA (Fig. 4A, lane 2). Addition of up to 0.63 μM H-NS to this complex had no significant effect on the SlyA:P*hlyE* complex (Fig. 4A, lanes 3–5), but mobility was increased in the presence of 1.25 and 2.5 μM H-NS (Fig. 4A, lanes 6 and 7). This increase in mobility in the presence of H-NS indicates the formation of a SlyA:H-NS:P*hlyE* complex (note that in comparison with SlyA:P*hlyE*, the H-NS:P*hlyE* complex migrates significantly further; Fig. 4B, lane 2). In the reverse experiment, increasing amounts of SlyA were added to a complex of P*hlyE* and 1.25 μM H-NS. The mobility of H-NS:P*hlyE* was not affected by addition of up to 0.63 μM SlyA (Fig. 4B, lanes 2–5), but was significantly altered after addition of 1.25 μM or 2.5 μM SlyA (Fig. 4B, lanes 6 and 7). The simplest explanation for these data is that initial formation of a SlyA:P*hlyE* complex prevents binding of H-NS when H-NS is present at low concentrations. When a critical concentration of H-NS is reached, a SlyA:H-NS:P*hlyE* complex forms with an altered structure, as indicated by the changes in the DNase I footprint and EMSA mobility. The DNase I footprints (Fig. 2, lane 8) indicate that H-NS can ultimately displace SlyA. However, when the SlyA concentration increases relative to that of H-NS, the H-NS:P*hlyE* complex is replaced by a SlyA:P*hlyE* complex via a SlyA:H-NS:P*hlyE* intermediate. The switches between these different nucleoprotein complexes appear to occur over narrow concentration ranges, suggesting that at critical thresholds a ‘see-saw’ mechanism operates in which SlyA antagonizes H-NS interaction and H-NS antogonizes SlyA binding at P*hlyE*.

The effect of adding SlyA or H-NS to P*hlyE* in the presence of RNAP was then tested. Addition of up to 2.5 μM SlyA did not affect the mobility of the RNAP:P*hlyE* complex (Fig. 4C, lanes 2–7). Although it is not known at this stage whether both RNAP and SlyA are bound at P*hlyE* simultaneously, the mobility of a mixture of P*hlyE*, RNAP and SlyA was lower than SlyA:P*hlyE* alone (Fig. 4C, lanes 7 and 8), indicating that addition of SlyA did not interfere with the binding of RNAP to P*hlyE*. However, the RNAP:P*hlyE* complex was severely affected by the addition of H-NS (Fig. 4D). As the H-NS concentration was increased, the proportion of RNAP:P*hlyE* complex was reduced and the proportion of H-NS:P*hlyE* was increased (Fig. 4D, lanes 2–7), suggesting that H-NS replaces RNAP at P*hlyE* in a concentration-dependent manner. Thus, the EMSA data supported the hypothesis that H-NS negatively regulates P*hlyE* by promoter occlusion. In contrast, SlyA did not prevent RNAP binding at P*hlyE*, but did inhibit H-NS binding.

### SlyA relieves H-NS-dependent repression of *hlyE* transcription *in vitro*

**Fig. 5:**
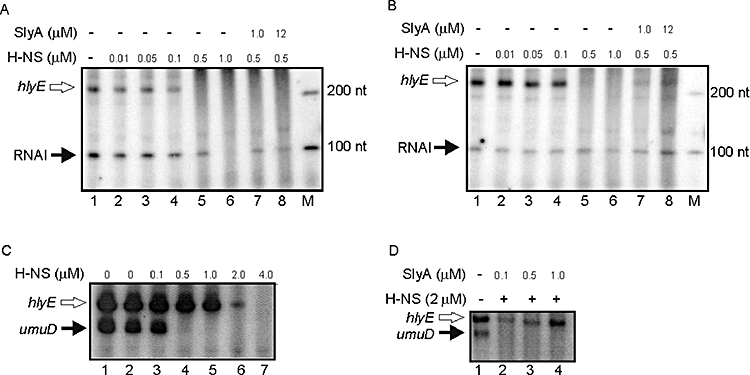
H-NS-dependent repression and SlyA-dependent derepression of *hlyE* transcription. Single-round *in vitro* transcription assays were carried out with plasmid pGS1886 containing the *hlyE* promoter (−474 to +222 relative to the transcript start) and RNAI control gene. A and B. Linearized pGS1886 DNA (20 ng) was incubated with 1 unit of RNAP and varying amounts of H-NS and SlyA as indicated in μM at (A) 25°C and (B) 37°C. Radiolabelled transcripts (lanes 1–8) and RNA markers (lane M) were separated on 4% acrylamide/1× TBE gels and analysed by autoradiography. White block arrows, 222 nt *hlyE* transcript; black arrows, 100 nt RNAI control transcript. C. Inhibition of transcription *in vitro* from P*hlyE* and P*umuD* by H-NS. A PCR-amplified DNA fragment extending from −474 to +222 relative to the *hlyE* transcript start containing the *hlyE* promoter and the promoter for the divergently transcribed *umuD* gene was used in *in vitro* transcription assays in the presence of increasing concentrations of H-NS as described above. The *hlyE* (222 nt) and *umuD* (202 nt) transcripts are indicated. D. Restoration of *hlyE* transcription by SlyA. *In vitro* transcription assays as described in (C) but in the presence of both H-NS (2 μM) and increasing amounts of SlyA. The *hlyE* (222 nt) and *umuD* (202 nt) transcripts are indicated.

The footprinting and EMSA data show that different nucleoprotein complexes are formed at P*hlyE* depending on the relative concentrations of H-NS and SlyA through competition for overlapping binding sites upstream and downstream of the *hlyE* transcript start site. This suggested a regulatory mechanism whereby increased intracellular concentrations of SlyA remodel H-NS binding at P*hlyE* to relieve H-NS-mediated repression by allowing RNAP to access the promoter. Plasmid pGS1886 contains the P*hlyE* and the 5′-end of the *hlyE* gene extending from −474 to +222 relative to the transcript start site. It was predicted that addition of H-NS would repress production of *hlyE* mRNA in an *in vitro* transcription reaction using pGS1886, and that addition of SlyA would modulate the effect of H-NS and allow *hlyE* transcription. At 25°C RNAP-induced expression of a *hlyE* transcript of 222 nt (Fig. 5A, lane 1) was abolished when 0.5 μM H-NS was added to the reaction (Fig. 5A, lane 5). In a mixture of RNAP and 0.5 μM H-NS supplemented with 1 μM or 12 μM SlyA, the *hlyE* transcript was still repressed at 25°C (Fig. 5A, lanes 7 and 8). However, at 37°C, addition of 1 μM or 12 μM SlyA partially relieved the repression caused by the presence of 0.5 μM H-NS (Fig. 5B, compare lanes 5 and 6 with lanes 7 and 8). Although reproducible, the enhancement in the abundance of the *hlyE* transcript when SlyA was added to the P*hlyE*:H-NS complex was somewhat disappointing under the conditions used for the experiments in Fig. 5A and B. Using an equivalent supercoiled template in place of the linearized DNA used for the experiments in Fig. 5A and B failed to improve SlyA-mediated inhibition of SlyA repression under the same conditions (not shown). However, a greater effect was observed when a PCR product (−474 to +222 relative to the *hlyE* transcript start) that contained both P*hlyE* and the promoter of the divergently transcribed *umuD* gene was used in the *in vitro* transcription reactions. Addition of 2 μM H-NS repressed both *hlyE* and *umuD* transcription (Fig. 5C). Therefore, 2 μM H-NS was used in SlyA titration experiments. At low SlyA:H-NS ratios both P*hlyE* and P*umuD* were still repressed (Fig. 5D, lanes 2 and 3). However, at a higher concentration of SlyA, transcription of *hlyE* was specifically restored (Fig. 5D, lane 4). In contrast to P*hlyE*, *umuD* was repressed whenever H-NS was present, showing that SlyA specifically antagonizes H-NS repression of P*hlyE*.

## Discussion

Although it has been previously demonstrated that the introduction of *slyA* on a multicopy plasmid confers a haemolytic phenotype on *E. coli* by enhancing expression of the haemolysin E gene, it is possible that this could be an artefactual effect of SlyA overexpression that is not normally seen in wild-type bacteria (Ludwig *et al*., 1995). However, in this study, *E. coli* mutants with lesions in chromosomal *slyA* showed significantly less expression of a chromosomal *hlyE–lacZ* fusion in aerobic, glucose-supplemented cultures, suggesting that, at least in these conditions, SlyA is involved in upregulation of *hlyE* expression in the *E. coli*.

It has previously been shown that H-NS binds to P*hlyE* in regions upstream (H-NS I) and downstream (H-NS II) of the transcript start site, and that a SlyA binding site overlaps H-NS I (Westermark *et al*., 2000; Wyborn *et al*., 2004b). In addition, both FNR and CRP contribute to regulation of *hlyE* expression (Green and Baldwin, 1997; Ralph *et al*., 1998; Westermark *et al*., 2000). FNR and CRP are ∼50 kDa proteins that recognize related inverted repeat sequences (Scott *et al*., 2000). A sequence in P*hlyE* (^−69^TTTGATATTTATCATA^−54^) more closely resembles the FNR consensus (NTTGATNNNNATCAAN) than the CRP consensus (TGTGANNNNNNTCACA), and *in vivo* transcription studies revealed that although FNR occupies P*hlyE* more frequently than CRP, the latter is a more efficient activator of *hlyE* expression (Wyborn *et al*., 2004b). These data suggested that *hlyE* expression is activated by FNR and CRP, repressed by H-NS, and that H-NS-mediated transcriptional silencing is relieved by SlyA. In this study *in vivo* and *in vitro* analyses show that SlyA competes with H-NS for binding sites located upstream and downstream of the *hlyE* transcriptional start site, revealing that SlyA can directly antagonize H-NS-mediated silencing of *hlyE* expression.

Previous DNase I protection assays using a shorter region of P*hlyE* than used here identified a SlyA footprint extending from −70 to −30 relative to the *hlyE* transcript start site (Wyborn *et al*., 2004b). Within this region are two potential 12 bp SlyA-recognition motifs, SlyA Ia, ^−61^**TTA**T**CA**TAT**TAA**^−50^, and SlyA Ib, ^50^A**TAG**A**AA**TAA**A**G^−39^ (consensus-matching bases in bold). In SlyA Ia, 8 out of 12 base pairs match the SlyA-recognition consensus (**TTAGCAAGCTAA**) compared with only 6 out of 12 base pairs in SlyA Ib. In this study we identified a second region of P*hlyE* extending from +34 to +109 that is protected by SlyA. In this region, SlyA II contains potential SlyA-recognition motifs SlyA IIa (^+47^**TTA**TAT**A**TT**TAA**^+58^, consensus match 7/12), SlyA IIb (^+56^**T**A**A**AG**A**G**GC**G**AA**^+67^, 7/12), SlyA IIc (^+71^**TTA**TG**A**CTGA**AA**^+82^, 6/12) and SlyA IId (^+85^G**T**G**GCA**GATA**AA**^+96^, 6/12).

The downstream position of the SlyA II site is an unusual location for typical bacterial transcriptional activators. However, it has been shown that some other *S.* Typhimurium genes that are positively regulated by SlyA contain SlyA binding sites downstream from the transcript start site, including *pagC* and *ugtL* (Shi *et al*., 2004; Navarre *et al*., 2005). Consistent with our observations at P*hlyE*, it has been suggested that SlyA binding to such sites remodels the local nucleoprotein structure, counteracting the effect of negative regulators such as H-NS, and clearing the way for classical transcriptional activators such as PhoP, or as in the case of *hlyE*, FNR and CRP (Ellison and Miller, 2006).

**Fig. 6:**
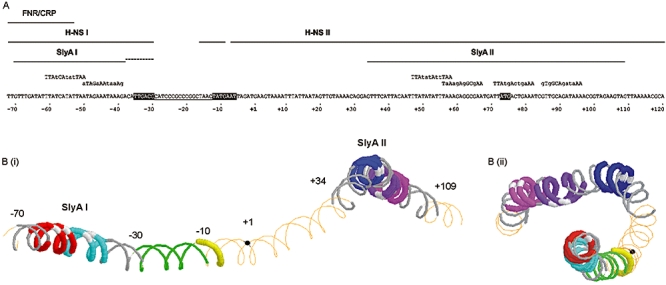
Nucleotide sequence and predicted three-dimensional structure of the *hlyE* promoter region. A. Position of H-NS and SlyA binding sites in the *hlyE* promoter. DNA sequence shown from −72 to +120 relative to the *hlyE* transcript start site (white arrow). Putative −35 (TTGACG) and heptameric −10 (TATGAAT) elements and the HlyE translational start codon ATG at +73 are highlighted in black boxes. The GC-rich spacer is underlined. Regions protected by H-NS and SlyA in footprint assays (H-NS I, H-NS II, SlyA I and SlyA II), and the FNR/CRP binding site described by Westermark *et al*. (2000) are indicated by lines above the sequence. The dotted line indicates the part of the SlyA I site that is protected only at higher concentrations of SlyA. Potential palindromic SlyA recognition sites are shown above the DNA sequence with residues matching the consensus (TTAGCAAGCTAA) in upper case: SlyA Ia (TTAtCAtatTAA), SlyA Ib (aTAGaAAtaaAg), SlyA IIa (TTAtatAttTAA), SlyA IIb (TaAagAGCgAA), SlyA IIc (TTAtgActgaAA) and SlyA IId (gTgGCAgataAA). B. Three-dimensional representation of the *hlyE* promoter obtained from DNA curvature prediction with the Model.it server. The positions of the 12 bp SlyA-recognition sequences SlyA Ia, Ib and SlyA IIa, IIc and IId are shown in red, cyan and blue, purple and magenta respectively. White segments highlight the minor groove and the face of the DNA helix to which SlyA potentially binds. The extended SlyA-protected regions protected by SlyA in DNase I footprinting at SlyA I and SlyA II are coloured grey. The GC-rich spacer, −10 element and +1 transcript start site are shown in green, yellow, green and black respectively. Projection (i) was rotated 45° around the +1 site in the *z*-axis orientation to give projection (ii).

P*hlyE* is AT-rich and previous bending predictions have shown intrinsic curvature and sharp bends in the *hlyE* gene (Westermark *et al*., 2000). Our analysis using the Model.it server gave similar results, showing the *hlyE* translational start site (+73 relative to the transcription start) is approximately located at an apex of curvature in the region (Fig. 6B). P*hlyE* is thus suitable for interaction with H-NS, which is known to bind preferentially to AT-rich curved DNA (Bracco *et al*., 1989). In addition, H-NS binding further increases DNA curvature (Rimsky, 2004). In contrast to the H-NS I and H-NS II binding sites upstream and downstream of the transcript start site, there is no H-NS protection in the region of the GC-rich spacer between the −35 and −10 elements (Fig. 6A). A similar H-NS protection pattern has been seen in the promoter of the glycine-betaine transport operon *proU* (Lucht and Bremer, 1994).

Previous investigations highlighted the importance of the −35 to −10 region of P*hlyE* for derepression by SlyA (Ludwig *et al*., 1999). It contains the unusual −10 element TATGAAT, and it is likely that affinity of a RNAP sigma factor for this heptamer is less than that of a typical TATAAT hexamer. Furthermore, a reduction in the GC content of the GC-rich spacer diminished the capability of SlyA to activate *hlyE* expression (Ludwig *et al*., 1999). The observations reported here suggest that lowering the GC content of the spacer could increase DNA curvature and H-NS affinity in this region, increasing the stability of the DNA:H-NS complex, and preventing derepression by SlyA.

**Fig. 7:**
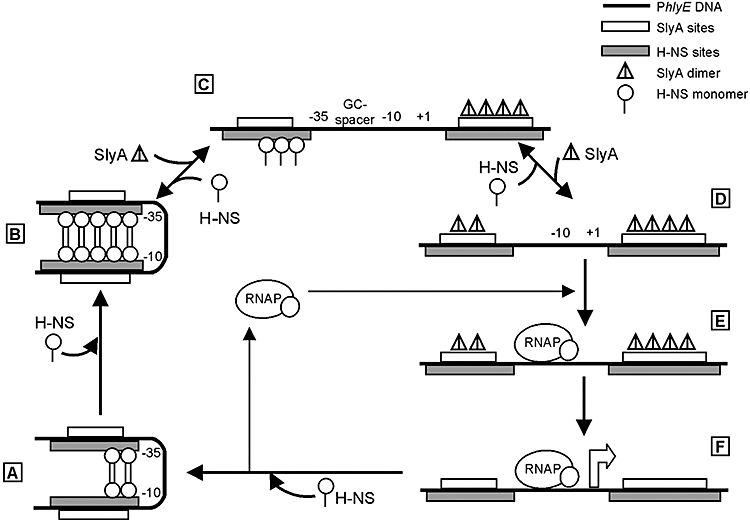
Model of a SlyA/H-NS regulatory loop at the *hlyE* promoter. At low concentrations of SlyA, H-NS can bind initially at AT-rich nucleation sites H-NS I and H-NS II. H-NS binding increases the DNA curvature enabling looping of the promoter and the formation of H-NS-dimer bridges connecting the H-NS I and II sites (A). The zone of H-NS-binding then extends by oligomerization, preventing *hlyE* transcription by promoter occlusion (B). When the concentration of SlyA increases, H-NS is displaced by the binding of SlyA first to the higher-affinity SlyA II site (C), and then to the SlyA I site (D). This section of the model is reversible, because when the relative concentration of H-NS increases H-NS is able to displace SlyA. Thus, P*hlyE*‘see-saws’ between an H-NS dominated nucleoprotein complex (A and B), which silences expression, and a SlyA dominated complex, which promotes expression (D). The remodelled SlyA:DNA complex (D) maintains the −35, −10 and GC-rich spacer elements in a conformation that allows RNAP to bind P*hlyE* (E) and transcription (indicated by the open curved arrow) of *hlyE* can then proceed (F). When H-NS concentrations increase relative to SlyA, SlyA and RNAP binding is inhibited and *hlyE* transcription is silenced (A and B).

The experimental data reported here are consistent with gene silencing models in which the curved DNA to which H-NS initially binds is located upstream or downstream of the promoter that is to be repressed (Rimsky, 2004). It was recently suggested that H-NS dimers form DNA:H-NS:DNA bridges that trap RNAP at promoters (Dorman, 2004; 2007; Dame *et al*., 2005; 2006). In this study it is shown that transcription of *hlyE in vitro* is repressed by the addition of H-NS alone and it is likely that this is caused by H-NS dimers initially bridging between the H-NS I and H-NS II sites. Therefore, it is suggested that H-NS first binds at AT-rich regions up- and downstream of the *hlyE* transcript start site (Wyborn *et al*., 2004b; Fig. 7, A). This binding increases the curvature of DNA allowing formation of a DNA:H-NS:DNA bridge. The zone of H-NS protection then extends by oligomerization, ‘zipping-up’ the up- and downstream regions of P*hlyE*, and silencing transcription by trapping or excluding RNAP (Fig. 7, B). A recent investigation of DNA:H-NS interaction kinetics showed that a DNA:H-NS:DNA complex can be unzipped by a force of ∼7 pN at a rate of 70 bp s^−1^, while RNAP can exert forces of up to 25 pN (Wang *et al*., 1998; Dame *et al*., 2006). The H-NS:DNA interaction can therefore be easily overcome in the right conditions, which SlyA may help to establish by allowing dynamic re-organization of promoter complexes in response to different environmental stimuli. The footprinting analysis shows that binding sites for SlyA overlap the AT-rich H-NS-binding sequences and that SlyA affects the ability of H-NS to bind to P*hlyE*, probably by binding first to the higher-affinity SlyA II site to form an intermediate complex SlyA:H-NS:P*hlyE* in which the H-NS dimer interactions bridging H-NS II and H-NS I are disrupted (Fig. 7, C and D). This SlyA-remodelled nucleoprotein complex is capable of accommodating RNAP allowing *hlyE* expression (Fig. 7, E and F). When the intracellular concentration of SlyA decreases, or the concentration of H-NS increases, H-NS is able to displace both SlyA and RNAP silencing *hlyE* expression (Fig. 7, A and B). Thus, in the proposed regulatory model the relative concentrations of SlyA and H-NS determine the type of nucleoprotein complex formed at P*hlyE* and when SlyA is dominant it inhibits binding of H-NS and, in combination with the GC-rich spacer region, maintains the topology of the promoter in a conformation that enables RNAP to begin *hlyE* transcription. While SlyA-mediated antagonism of H-NS-mediated silencing has been suggested previously it is now shown that H-NS can interfere with SlyA binding. Thus, SlyA and H-NS operate a ‘see-saw’ mechanism, switching between SlyA- and H-NS-based nucleoprotein complexes to either promote (SlyA) or silence (H-NS) *hlyE* expression. This mechanism has a number of parallels with the recently reported H-NS antagonism by VirB at the *Shigella flexneri iscB* promoter (Turner and Dorman, 2007). Like SlyA at P*hlyE*, VirB has no positive effect on *iscB* expression in the absence of H-NS, but VirB alters the structure of P*iscB* in the region occupied by H-NS and this requires a DNA sequence located downstream of the *iscB* transcript start (Turner and Dorman, 2007).

Another related mechanism to that proposed here has been described for the *virF* virulence gene promoter of *S. flexneri*, where interaction between H-NS proteins bound to two segments of AT-rich DNA facilitates promoter looping and repression of *virF* transcription (Falconi *et al*., 1998). However, the DNA:H-NS interaction is also dependent on environmental conditions including temperature, which alters the degree of DNA curvature in the *virF* promoter leading to the displacement of H-NS due to a shift in the location of the centre of the bend (Prosseda *et al*., 2004). *In vivo* and *in vitro* transcription assays suggest that an increase in temperature alone cannot relieve repression of *hlyE* by H-NS (Table 1, Fig. 5). However, SlyA counteracts H-NS-mediated repression results in derepression of *hlyE* transcription *in vitro* at 37°C, but not at 25°C. This may be due to increased stability of H-NS:P*hlyE* complex at lower temperatures. Although derepression by SlyA *in vitro* is temperature-dependent this did not appear to be the case *in vivo* (Table 1), suggesting there are additional factors at play *in vivo*.

SlyA is closely related to the *Y. pseudotuberculosis* regulator RovA, which regulates expression of the virulence factor invasin. The *inv* promoter has overlapping binding sites for RovA and H-NS, and RovA functions as an antirepressor of *inv* expression when H-NS is present (Heroven *et al*., 2004; Tran *et al*., 2005). RovA was also shown to activate *inv* transcription by direct contact with RNAP in the absence of H-NS. Although it is possible that it may activate some promoters directly, the addition of SlyA to RNAP did not increase the amount of *hlyE* transcription *in vitro* (data not shown). The level of expression of a chromosomal *hlyE–lacZ* fusion in an *E. coli hns slyA* double mutant was slightly greater than in an *hns* single mutant, suggesting that SlyA may have a small negative effect on *hlyE* transcription *in vivo* in the absence of H-NS, and active CRP and FNR (Table 1). A negative effect of SlyA on *hlyE* expression may be a consequence of the proximity of the promoter −35 element and the SlyA Ib recognition site (Fig. 6A). It has been shown that relief of H-NS-mediated *hlyE* silencing by SlyA is much less efficient in the absence of CRP as the *hlyE* promoter normally needs CRP or FNR to be activated to higher levels (Westermark *et al*., 2000; Wyborn *et al*., 2004b). This is consistent with our observation that in the absence of CRP and FNR, addition of SlyA only leads to partial derepression of the *hlyE* promoter *in vitro*.

Virulence factor production by bacteria is an energy-expensive process that is also likely to alert the defence mechanisms of host cells, and it is therefore vital that bacteria can tightly repress virulence genes, but also that the repression can be quickly reversed in response to appropriate conditions. Many bacterial virulence factors are acquired by horizontal gene transfer and it has been suggested that gene silencing by H-NS and H-NS-like proteins allows the newly acquired genes to be integrated into regulatory networks with little effect on the fitness of the bacterium (Doyle *et al*., 2007). However, to function as virulence factors the genes must be expressed when it is advantageous to do so. Consequently, the ability of SlyA to act as an H-NS antagonist is likely to be important for bacterial pathogenesis and evolution.

## Experimental procedures

### Bacterial strains, plasmids and microbiological methods

Relevant characteristics of bacterial strains, plasmids and oligonucleotides used are given in Table 2. The *slyA*::Tn*5* mutant FB21922, containing a transposon insertion at position +198 from the *slyA* start codon, was obtained from the Blattner laboratory (Kang *et al*., 2004). The mutation was transferred to *E. coli* M182 via P1*vir*-mediated transduction to create strain JRG5358, and also into *hns* mutant strain JRG4864 to produce a *hns slyA* double mutant. The *hlyE–lacZ* fusion from plasmid pGS1629 was transduced with phage λ into *E. coli* M182 and the *slyA*, *hns* and *hns slyA* mutants to produce single-copy chromosomal *hlyE–lacZ* reporter strains JRG5580, 5581, 5582 and 5583. Bacteria were grown in L broth (tryptone 10 g l^−1^; yeast extract 5 g l^−1^; NaCl 10 g l^−1^) at 20°C, 25°C or 37°C. Media were supplemented with ampicillin (Ap, 100 μg ml^−1^), tetracycline (Tet, 35 μg ml^−1^) kanamycin (Kn, 20 μg ml^−1^) or chloramphenicol (Cm, 20 μg ml^−1^) as appropriate. For β-galactosidase measurements, cultures were grown in 250 ml universal bottles containing either 10 ml (aerobic) or 250 ml (anaerobic) of l-broth with shaking at 250 r.p.m. and promoter activities were estimated according to Miller (1972). Standard methods for manipulation of DNA were followed (Sambrook and Russell, 2001). *E. coli* M182 genomic DNA was used as a template in all PCR reactions described below. To make the *in vitro* transcription plasmid pGS1886, SQ101 and SQ102 were used to amplify by PCR the *hlyE* promoter [−474 to +222 bp relative to the *hlyE* transcriptional start determined by Ludwig *et al*. (1999) and Westermark *et al*. (2000)]. The fragment, which also contained the first 174 bp of the divergently transcribed *umuD* coding region, was digested with EcoRI and HindIII at the restriction sites built into the oligos and ligated into EcoRI–HindIII-digested pRLG770 vector (Ross *et al*., 1990). To create the H-NS overexpression plasmid pGS1875, oligos SQ25 and SQ26 were used to amplify the 411 bp H-NS coding region. SQ25 contains an artificial BamHI site adjacent to the H-NS start codon, and SQ25 contains an engineered HindIII site overlapping the H-NS stop codon. The fragment was ligated with pGEX-KG vector that had been digested with BamHI and HindIII, creating a GST–H-NS fusion. To produce the *hlyE* promoter footprinting construct pGS2079, DNA was amplified using oligos SQ105 and SQ106 and digested at the engineered EcoRI and HindIII sites to produce a 244 bp fragment starting at −171 relative to the transcript start, and finishing at the *hlyE* translation start codon at +73. This was ligated into EcoRI–HindIII-digested pRLG770 and checked by DNA sequencing. Another construct for footprinting, pGS2135, containing a 393 bp *hlyE* promoter fragment from −171 to +222 was produced in a similar way using oligos SQ102 and SQ105 with ligation of the fragment into EcoRI–HindIII-digested pUC18. Plasmids were transformed into *E. coli* DH5α and other strains by standard methods (Sambrook and Russell, 2001).

**Table 2:** Bacterial strains, plasmids and oligonucleotides used in this study.

Strain or plasmid	Genotype or relevant characteristics	Source or reference
*E. coli*		
DH5α	Δ*lac*	Sambrook and Russell (2001)
M182	Δ*lac*	Busby *et al*. (1983)
BL21/λDE3	Protease-deficient strain used for protein expression	Studier (1975)
FB21922	M1655 *slyA*::Tn*5* mutant; Kn^R^	Kang *et al*. (2004)
JRG4864	M182 Δ*hns*; Cm^R^	Wyborn *et al*. (2004b)
JRG5329	M182 Δ*hns slyA*::Tn*5*; Cm^R^ Kn^R^	This work
JRG5358	M182 *slyA*::Tn*5*; Kan^R^	This work
JRG5580	M182 *hlyE–lacZ* single-copy chromosomal reporter fusion; Ap^R^	This work
JRG5581	M182 *hlyE–lacZ slyA*::Tn*5*; Ap^R^ Kn^R^	This work
JRG5582	M182 *hlyE–lacZ*Δ*hns*; Ap^R^ Cm^R^	This work
JRG5583	M182 *hlyE–lacZ slyA*::Tn*5*Δ*hns*; Ap^R^ Cm^R^ Kn^R^	This work
Plasmid		
pGEX-KG	Ap^R^ GST fusion expression vector	Guan and Dixon (1991)
pRLG770	Ap^R^ transcription plasmid with internal RNAI control gene	Ross *et al*. (1990)
pRW50	Low-copy-number Tet^R^*lac* reporter vector	Lodge *et al*. (1990)
pUC18	Ap^R^ high-copy-number cloning vector	Vieira and Messing (1991)
pUC118	Ap^R^ high-copy-number cloning vector	Vieira and Messing (1991)
pGS1482	GST–SlyA overexpression plasmid	Stapleton *et al*. (2002)
pGS1629	*hlyE–lacZ* reporter plasmid	Wyborn *et al*. (2004b)
pGS1875	GST–H-NS overexpression plasmid	This work
pGS1886	696 bp *hlyE* promoter fragment in pRLG770 for *in vitro* transcription	This work
pGS2079	242 bp *hlyE* promoter fragment in pRLG770 for DNA footprinting	This work
pGS2135	393 bp *hlyE* promoter fragment in pUC18 for DNA footprinting	This work
Oligonucleotide^a^		
SQ25	GTTT*GGATCC***ATG**AGCGAAGCACTTAAAATTCTG	
SQ26	ACAA*AAGC****TT*A**TTGCTTGATCAGGAAATCG	
SQ101	CCCC*GAATTC*ACCACTTGCTTTGACGAAG	
SQ102	AAAG*AAGCTT*AACTCTTTTATGGTTTC	
SQ105	CACGTG*GAATTC*TGGCGACGACGC	
SQ106	TTTC*AAGCTT*AATCATTCGCCTC	

a Engineered restriction sites in oligonucleotides are italicized and positions of start and stop codons of H-NS are shown in bold.

### Protein purification

SlyA and H-NS were overexpressed as GST fusion proteins in *E. coli* BL21/λDE3 transformed with pGS1482 and pGS1875 as described previously (Stapleton *et al.*, 2002). Thrombin (Sigma) was used to cleave GST tags and protein fractions were eluted from glutathione-Sepharose columns in GST buffer [5 mM Tris-Cl pH 8150 mM NaCl, 2.5 mM CaCl_2_ 0.1% (w/v) β-mercaptoethanol]. CRP was obtained from Steve Busby and RNAP holoenzyme was obtained from Epicentre.

### Electrophoretic mobility shift assays

The 244 bp *hlyE* promoter fragment was excised from pGS2079 by digestion with HindIII and EcoRI and radioactively labelled using Klenow fragment and 20 μCi ^32^P-labelled dATP (800 Ci mmol^−1^, Perkin Elmer). In a 20 μl reaction volume, 0.2 μg of labelled DNA was incubated at 25°C or 37°C for 15 min with 2 μl of 10× binding buffer (100 mM Tris-HCl pH 7.5, 10 mM EDTA, 50 mM DTT, 50% glycerol, 100 mM NaCl, 10 mM MgCl_2_) and varying amounts of RNAP, SlyA or H-NS as indicated. After addition of 2 μl of loading buffer (5% glycerol, 0.1% bromophenol blue) the reactions were separated using a 4% acrylamide, 1× TBE (100 mM Tris, 120 mM boric acid, 10 mM EDTA pH 8.0) gel. Gels were dried on Whatman paper and subjected to autoradiographic analysis.

### *In vitro* run-off transcription assays

Plasmid pGS1886 was digested with HindIII and 20 ng of the linearized plasmid DNA was incubated for 5 min at 37°C in a 21 μl reaction volume containing 40 mM Tris-acetate pH 7.9, 10 mM MgCl_2_, 1 mM DTT, 100 mM KCl, 100 μg ml^−1^ BSA and varying amounts of SlyA and H-NS as indicated. Single-round transcription reactions were incubated for 15 min at 37°C in the presence of 4 μl of a solution containing unlabelled and labelled nucleotide triphosphates [UTP at 50 μM; ATP, CTP and GTP at 1.2 mM; and 5 μCi ^32^P-labelled UTP (800 Ci mmol^−1^, Perkin Elmer)], 0.5 μg of heparin and 1 unit of RNAP holoenzyme (Epicentre). Reactions were terminated by adding 18 μl of Stop/Loading dye solution (95% formamide, 20 mM EDTA pH 8, 0.05% bromophenol blue, 0.05% xylene cyanol), and 15 μl of each reaction was loaded on a 4% acrylamide, 1× TBE gel, dried and analysed by autoradiography.

### DNase I footprinting

Plasmid pGS2079 or pGS2135 was digested with HindIII and the recessed 3′-OH terminus was filled in using Klenow fragment and 20 μCi ^32^P-labelled dATP as described above. The DNA was then digested with EcoRI to produce a radiolabelled 244 bp or 393 bp *hlyE* promoter fragments. In separate reactions the order of restriction digestion was reversed to label the fragment on the other strand. Following purification by agarose gel extraction 250 ng of DNA fragment was incubated at room temperature in a 50 μl final volume containing varying amounts of SlyA and H-NS as indicated and 25 μl of DNA binding buffer from the Promega Core Footprinting System. Following the formation of protein:DNA complexes 50 μl of a solution of 5 mM CaCl_2_ and 10 mM MgCl_2_ and 3 μl of 1 U μl^−1^ RQ1 DNase I were added. Reactions were terminated with Promega Stop Solution after 2 min, extracted with phenol:chloroform, precipitated with ethanol and vacuum dried. A Maxam–Gilbert G-track reaction was used as a calibration as described previously (Maxam and Gilbert, 1980). Dried reactions were re-suspended in 10 μl of loading buffer (80% formamide, 0.1% bromophenol blue, 0.1% xylene cyanol, 10% glycerol, 8 mM EDTA pH 8) for electrophoretic fractionation on a 6% polyacrylamide, 7 M urea gel in 1× TBE buffer, and subjected to autoradiographic analysis.

### DNA curvature prediction

The curvature of the *hlyE* promoter was analysed using the web-based Model.it prediction program at http://hydra.icgeb.trieste.it/dna/model_it.html (Vlahoviček *et al*., 2003) and the resulting helix co-ordinates were displayed and visualized using RASMOL (Sayle and Milner-White, 1995).
